# MACMIC Reveals A Dual Role of CTCF in Epigenetic Regulation of Cell Identity Genes

**DOI:** 10.1016/j.gpb.2020.10.008

**Published:** 2021-03-05

**Authors:** Guangyu Wang, Bo Xia, Man Zhou, Jie Lv, Dongyu Zhao, Yanqiang Li, Yiwen Bu, Xin Wang, John P. Cooke, Qi Cao, Min Gyu Lee, Lili Zhang, Kaifu Chen

**Affiliations:** 1 Center for Bioinformatics and Computational Biology, Department of Cardiovascular Sciences, Institute for Academic Medicine, Houston Methodist Research Institute, Houston, TX 77030, USA; 2 Center for Cardiovascular Regeneration, Department of Cardiovascular Sciences, Institute for Academic Medicine, Houston Methodist Research Institute, Houston, TX 77030, USA; 3 Department of Cardiothoracic Surgeries, Weill Cornell Medical College, Cornell University, New York, NY 10065, USA; 4 Houston Methodist Institute for Academic Medicine, Houston Methodist Research Institute, Houston, TX 77030, USA; 5 Basic and Translational Research Division, Department of Cardiology, Boston Children's Hospital, Boston, MA 02115, USA; 6 Department of Pediatrics, Harvard Medical School, Boston, MA 02115, USA; 7 Department of Urology, Feinberg School of Medicine, Northwestern University, Chicago, IL 60611, USA; 8 Robert H. Lurie Comprehensive Cancer Center, Feinberg School of Medicine, Northwestern University, Chicago, IL 60611, USA; 9 Department of Molecular and Cellular Oncology, The University of Texas MD Anderson Cancer Center, Houston, TX 77030, USA

**Keywords:** Mutual information, Correlation, CCCTC-binding factor, H3K27ac, H3K27me3

## Abstract

Numerous studies of relationship between epigenomic features have focused on their strong **correlation** across the genome, likely because such relationship can be easily identified by many established methods for correlation analysis. However, two features with little correlation may still colocalize at many genomic sites to implement important functions. There is no bioinformatic tool for researchers to specifically identify such feature pairs. Here, we develop a method to identify feature pairs in which two features have maximal colocalization minimal correlation (MACMIC) across the genome. By MACMIC analysis of 3306 feature pairs in 16 human cell types, we reveal a dual role of **CCCTC-binding factor** (CTCF) in epigenetic regulation of cell identity genes. Although super-enhancers are associated with activation of target genes, only a subset of super-enhancers colocalized with CTCF regulate cell identity genes. At super-enhancers colocalized with CTCF, CTCF is required for the active marker **H3K27ac** in cell types requiring the activation, and also required for the repressive marker **H3K27me3** in other cell types requiring repression. Our work demonstrates the biological utility of the MACMIC analysis and reveals a key role for CTCF in epigenetic regulation of cell identity. The code for MACMIC is available at https://github.com/bxia888/MACMIC.

## Introduction

As DNA sequencing data expand at an unprecedented speed, genomic (including epigenomic) data such as RNA-seq, ChIP-seq, and genome sequencing data can be conveniently collected from public databases. Each set of sequencing data is typically collected to investigate a genomic (including epigenomic) feature across the genome, *e.g.*, RNA-seq dataset to investigate the expression profile of all genes in a genome, and ChIP-seq dataset to investigate a histone modification or the binding of a transcription factor at individual sites across the genome. It is commonly recognized that the function of a genome cannot be fully understood by studying a single genomic feature. Many studies have shown that analysis of correlation between two genomic features has a strong potential to identify their regulatory relationship in an important biological process [1], [2]. For instance, a strong positive correlation between the binding intensity of a protein near individual genes and the expression level of these genes might help define the protein to be an activator of transcription [3]. By focusing on the correlation between the RNA expression and a histone modification, the roles of individual histone modifications in the activation or repression of transcription have also been recognized [4], [5], [6].

However, in many aspects of informatics, the representation of knowledge can be more efficient by using a combination of uncorrelated features [7]. In other words, highly correlated features often contain redundant information [8]. For example, whereas the dozens of pluripotent factors such as Oct4, Sox2, Klf4, and c-Myc, are all useful to predict genes expressed in stem cells [9], [10], [11], combining some pluripotent factors with endothelial lineage factors such as Lmo2 and Erg would add power to also predict genes expressed in endothelial cells; therefore, it can be more powerful using combined information from transcription factors with distinct functions, as opposed to an analysis using the transcription factors with similar effects on a shared set of target genes. More importantly, colocalization of low-correlation chromatin features may still happen in a biologically meaningful manner to implement important functions. For instance, the histone modifications H3K27me3 and H3K4me3 are known to be associated with repression and activation of transcription in differentiated cells, respectively [12]. As a result, they show negative correlation and often occur at different genes in somatic cell types [13]. However, these two markers lose the negative correlation and colocalize at a large set of genes in embryonic stem cells (ESCs) [14], [15], [16]. It is well known now that the colocalization of H3K27me3 and H3K4me3 in ESCs defines bivalent chromatin domains, which are functionally distinct from both the repressive domains associated with H3K27me3 and the active domains associated with H3K4me3. These bivalent chromatin domains play a unique role in ESCs to maintain a bivalent status of the lineage factors for individual somatic cell types [17], [18], [19]. Therefore, analyzing colocalization of two chromatin features with globally low correlation in a cell has the potential to reveal novel biological mechanisms. However, little is known yet about the biological implications of such colocalization for the other chromatin features beyond H3K4me3 and H3K27me3. Therefore, the community is in need of a robust method to identify and understand the biologically important colocalizations of uncorrelated chromatin features in a cell.

In this study, we utilized mutual information [20], [21], [22] as an indication for general correlation (relevance) between a pair of genomic features, and mathematically integrated it with the number of colocalizations between the features to define a score for maximal colocalization minimal correlation (MACMIC). The MACMIC score allows us to quantitatively prioritize the feature combinations that have large number of colocalizations but low correlation. We next performed a systematic analysis of MACMIC scores between chromatin features using 1522 datasets for histone modifications or the binding of chromatin proteins from ESCs as well as somatic cell types. Our analysis successfully recaptured the previously discovered bivalent domain in ESCs, and further revealed a key role for CCCTC-binding factor (CTCF) in the epigenetic regulation of cell identity genes.

## Method

### Data collection

The RNA-seq data and ChIP-seq data for transcription factors and histone modifications from human primary somatic cells, human ESCs (hESCs), and mouse ESCs (mESCs) were downloaded from Gene Expression Omnibus (GEO) database and Encyclopedia of DNA Elements (ENCODE) project website (https://www.encodeproject.org/) [23]. Processed annotated topologically associating domains and loops from human umbilical vein endothelial cells (HUVECs) were downloaded from GEO. Detailed information of datasets reanalyzed in this study is listed in Tables S1 and S2.

### Data processing and analysis

Human reference genome sequence (version hg19), mouse reference genome sequence (version mm9), and University of California Santa Cruz (UCSC) Known Genes were downloaded from the UCSC Genome Browser website [24]. Transcripts per kilobase million (TPMs) of RNA-seq from ENCODE were directly downloaded from ENCODE project. For GEO datasets, RNA-seq raw reads were mapped to the human genome (version hg19) using TopHat (version 2.1.1) with default parameter values. The expression value for each gene was determined by the Cuffdiff function in Cufflinks (version 2.2.1) with default parameter values.

For ChIP-seq data, reads were first mapped to reference genome by Bowtie (version 1.1.0). Peak calling and generation of .wig file were performed by Dynamic Analysis of Nucleosome and Protein Occupancy by Sequencing (DANPOS; version 2.2.3). Bigwig was generated using the tool WigToBigWig, which was downloaded from the ENCODE project website (https://www.encodeproject.org/software/wigtobigwig/) [23]. Then bigwig file was submitted to the UCSC Genome Browser (https://genome.ucsc.edu) to visualize the ChIP-seq signal at each base pair [24], [25]. The average density plots of epigenetic marks in promoter region around transcription start site (TSS) were plotted using the Profile function in DANPOS (version 2.2.3). Heatmap was plotted using Morpheus (https://software.broadinstitute.org/morpheus). *P* values of boxplots were calculated with a two-sided Wilcoxon test. For the regulation network, we used CellNet method [26] to define the network and downloaded the network nodes (genes), edges, and value of closeness between nodes from CellNet website (http://cellnet.hms.harvard.edu/). As the gene number will affect the percentage and *P* value of overlap between gene groups, we used the same number of top genes from each group to avoid this effect. Because the genes associated with broad H3K4me3 was reported to be around 500 in each cell type [27], we used this number of genes for each gene group.

### Integrated analysis of two chromatin features

For individual markers, the ranking of genes was based on the width of individual markers on the gene promoter region (upstream 3 kb of TSS to downstream 10 kb of TSS). For the ranking of genes based on the colocalization of two chromatin features, the rank product of two individual markers was calculated first. We defined rank product as $\mathrm{RP}=\sqrt{\prod_{i=1}^{n}r_{1,i}*r_{2,i}}$ , where the $r_{1,i}$ is the rank of width for the first marker, the $r_{2,i}$ is the rank of width for the second marker. Then if no colocalization of these two chromatin markers was detected in the gene promoter region, the gene was being removed from the ranking. A colocalization of two chromatin markers at a specific genomic locus was defined by requiring at least 1-bp overlap. To measure the colocalization level of two chromatin markers, we calculated the total number of genomic loci that display overlap of these two chromatin markers across whole genome. Afterward, the genes associated with the colocalization of these two chromatin features were ranked based on the rank product of individual features. For a fair comparison, each group defined by broad H3K4me3, broad H3K27ac, broad H3K27me3, colocalization of broad H3K4me3 and broad H3K27me3, or colocalization of broad H3K4me3 and broad H3K27ac contained only the top 500 genes. GO term pathway analysis was performed by the web portal (http://geneontology.org/) [28].

### CTCF-associated super-enhancers

CTCF ChIP-seq datasets were processed as previously described. Peaks with height larger than upper quartile of peak height values were defined as high-confidence CTCF peaks. Super-enhancers were defined as previous defined [29], and then super-enhancers were categorized into two categories based on the existence of high-confidence CTCF peaks within super-enhancers. Super-enhancers with high-confidence CTCF peaks were named as CTCF-associated super-enhancers (CSEs). Super-enhancers without high-confidence CTCF peaks were named as other super-enhancers (OSEs).

### Simulation of association between CTCF and enhancers

For each group of typical enhancers, each typical enhancer was randomly matched to a super-enhancer, and then typical enhancers were enlarged towards two directions until they had the same size as super-enhancers. Associations of CTCF with super-enhancers, typical enhancers, and enlarged typical enhancers were calculated based on the overlapping events between the two different epigenetic markers.

### Mutual information of two genomic features

To calculate MACMIC score, we first calculated mutual information that is a widely used measure of the mutual dependence between two variables. A large mutual information value will indicate strong correlation that can be either positive or negative, and either linear or nonlinear. The rationale to use mutual information as an indication for correlation is that mutual information is more general than other methods such as linear correlation. Mathematically, mutual information is calculated by following equation:

$$I\left(X;Y\right)=H\left(X\right)+H\left(Y\right)-H\left(X,Y\right)$$

where *X* and *Y* represent the peak width from two different chromatin features, and *I*(*X*;*Y*) is the mutual information of *X* and *Y*. *H*(*X*) and *H*(*Y*) are the marginal entropies, and *H*(*X*,*Y*) is the joint entropy of *X* and *Y.* Entropies are calculated by the following equation:

$$H\left(X\right)=-\sum_{i=1}^{n}P\left(x_{i}\right)logP\left(x_{i}\right)$$

where *n* is the total gene number, and *P*(*x_i_***)** is the probability by which the total signal of a given genomic marker is *x_i_* in the promoter region of gene *i*. To calculate *H*(*X*), we focused on the promoter region from 3 kb upstream to 10 kb downstream of TSS. For a promoter that has multiple ChIP-seq peaks, we calculated the total signal that is the sum of signals in these peaks. The Selector function in DANPOS was used to map peaks to promoters. And we used Poisson distribution to calculate the probability of the observed ChIP-seq signal in a given promoter region [27]. To calculate the joint entropy of two genomic features, we used the following equation:

$$H\left(X,Y\right)=-\sum_{i=1}^{n}P\left(x_{i},y_{i}\right)logP\left(x_{i},y_{i}\right)$$

where *n* is the total gene number, and *P*(*x_i_*,*y_i_*) is the joint probability that the total signals of the first and second markers are *x_i_* and *y_i_*, respectively, in the promoter region of gene *i*.

### Regression model of genomic feature pairs

Theoretically, two features that have a small mutual information value tend to have no or a small number of colocalizations, whereas a large number of colocalizations are often associated with a large mutual information value. However, it is still unknown whether the colocalization of two histone modifications could identify genes that were not effectively identified by each of the two modifications. We first built a linear regression model to quantitively analyze the relationship between the mutual information value and the number of colocalizations. We used the least square method to estimate the parameters of the linear regression model. The data of the mutual information value and the number of colocalizations were calculated from 225 feature pairs which are derived from 6 chromatin features in 15 human primary somatic cell types (Table S1).

### Calculation of MACMIC score

**Figure 1:**
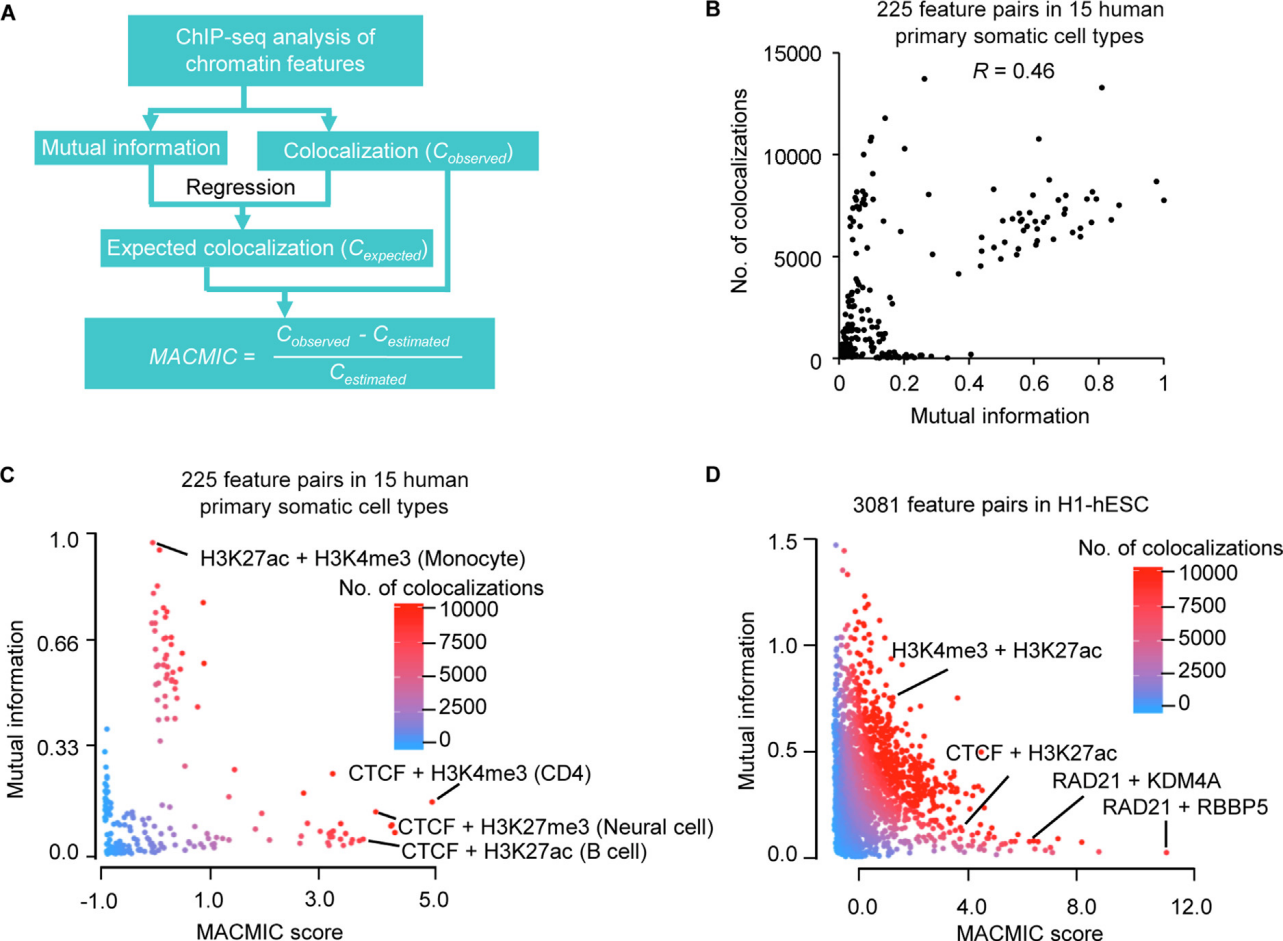
**The MACMIC method to define mutual information redundancy of colocalizations between genomic features** **A.** The workflow to calculate the MACMIC score. **B.** Scatter plot to show mutual information value and the number of colocalizations for each of 225 feature pairs derived from 6 features that form 15 combinations with each other in each of 15 human primary somatic cell types. **C.** Scatter plot to show MACMIC score and mutual information value for each of 225 feature pairs derived from 6 features that form 15 combinations with each other in each of 15 human primary somatic cell types. Color scale indicates the number of colocalizations between each pair of features. **D.** Scatter plot to show MACMIC score and mutual information value for each pair of features. 3081 feature pairs derived from 80 features in H1-hESC were plotted. Color scale indicates the number of colocalizations between each pair of features. MACMIC, maximal colocalization but minimal correlation; H1-hESC, human embryonic stem cell line H1; CTCF, CCCTC-binding factor; KDM4A, Lysine-specific Demethylase 4A; RBBP5, RB Binding Protein 5.

We developed MACMIC to prioritize feature pairs that have minimal correlation but a maximal number of colocalizations. A flowchart of MACMIC is presented in Figure 1A. Considering the penalty of high-correlation feature pairs, MACMIC score is calculated by the following equation:

$$\mathrm{MACMIC}=\frac{{C_{\mathrm{observed}}-C}_{\mathrm{expected}}}{C_{\mathrm{expected}}}$$

where *C* represents the number of colocalizations of two chromatin features which is counted by the number of overlapping events. The *P* value for each term tests the null hypothesis that the residual is equal to zero. A low *P* value (< 0.05) indicates that for a specific value of mutual information, the feature combinations have a significant higher colocalization than the estimated colocalization on the genome.

## Results

### Calculation of colocalization of globally low-correlation chromatin features

We first tested whether the colocalization of two histone modifications could identify genes that were not effectively identified by each of the two modifications. We performed the analysis for H3K4me3 and H3K27ac that had strong correlation across the genome (Figure S1A) and compared it to the analysis for H3K4me3 and H3K27me3 that had little correlation across the genome (Figure S1B) in hESC line H1 (H1-hESC). We recently revealed that the top 500 genes associated with broad H3K4me3 were enriched with tumor suppressor genes [27]. For a fair comparison, we retrieved the top 500 genes associated with broad H3K27ac and the top 500 genes associated with broad H3K27me3. There were 288 (57.6%) genes associated with both broad H3K4me3 and broad H3K27ac (Figure S1C). In contrast, there was no gene associated with both broad H3K4me3 and broad H3K27me3 (Figure S1D). To further explore the potential colocalization between H3K4me3 and H3K27me3, we defined the top 500 genes by the rank product of H3K4me3 width and H3K27me3 width (colocalization of broad H3K4me3 and broad H3K27me3) (Figure S1E). We also defined the top 500 genes by the rank product of H3K4me3 width and H3K27ac width (colocalization of broad H3K4me3 and broad H3K27ac) (Figure S1E). For the genes associated with colocalization of broad H3K4me3 and broad H3K27ac, only 7 genes were not captured by broad H3K4me3 or broad H3K27ac (Figure S1C). However, for the genes associated with colocalization of broad H3K4me3 and broad H3K27me3, 421 (84.2%) genes were not captured by broad H3K4me3 or broad H3K27me3 (Figure S1D). Further, for the 2168 pathways significantly enriched in genes associated with colocalization of broad H3K4me3 and broad H3K27me3, 1404 pathways showed no significant enrichment in genes associated with broad H3K4me3 or broad H3K27me3 (Figure S1F). These pathways were mainly related to somatic cell lineage specification (Figure S1G), which agreed with the reported role of bivalent domains. These results suggested that colocalization of globally low-correlation features in a cell could be associated with unique biological implications that were not associated with each of these features.

### MACMIC as a new method to identify association between chromatin features

We next developed the MACMIC algorithm to detect feature pairs in which the two associated features have large number of colocalizations but low global correlation across the genome (see Method). We performed MACMIC analysis of 6 features, which formed 15 pairs with each other in each cell type and thus formed 225 feature pairs in 15 human primary somatic cell types (Table S3). Most feature pairs displayed a positive correlation between the mutual information value and the number of colocalizations (Spearman correlation coefficient 0.46) (Figure 1B). Similar results were observed by replacing mutual information with absolute value of correlation coefficient or principal component analysis (PCA) value (Figure S2A and B). However, there were a few feature pairs that displayed a large number of colocalizations but a small mutual information value (Figure 1B). We calculated the MACMIC scores for the 225 individual feature pairs and found that the large MACMIC scores effectively prioritized feature pairs that possessed large number of colocalizations but weak correlations across the genome (Figure 1C). We observed the similar results by replacing mutual information with absolute value of correlation coefficient or PCA value as well (Figure S2C and D). We further tested our MACMIC analysis method on 3081 feature pairs derived from 80 chromatin features in H1-hESC. Our results again indicated that MACMIC successfully prioritized the feature pairs with minimal mutual information but substantial colocalizations (Figure 1D).

### MACMIC identifies a unique association of **CTCF** with super-enhancers

**Figure 2:**
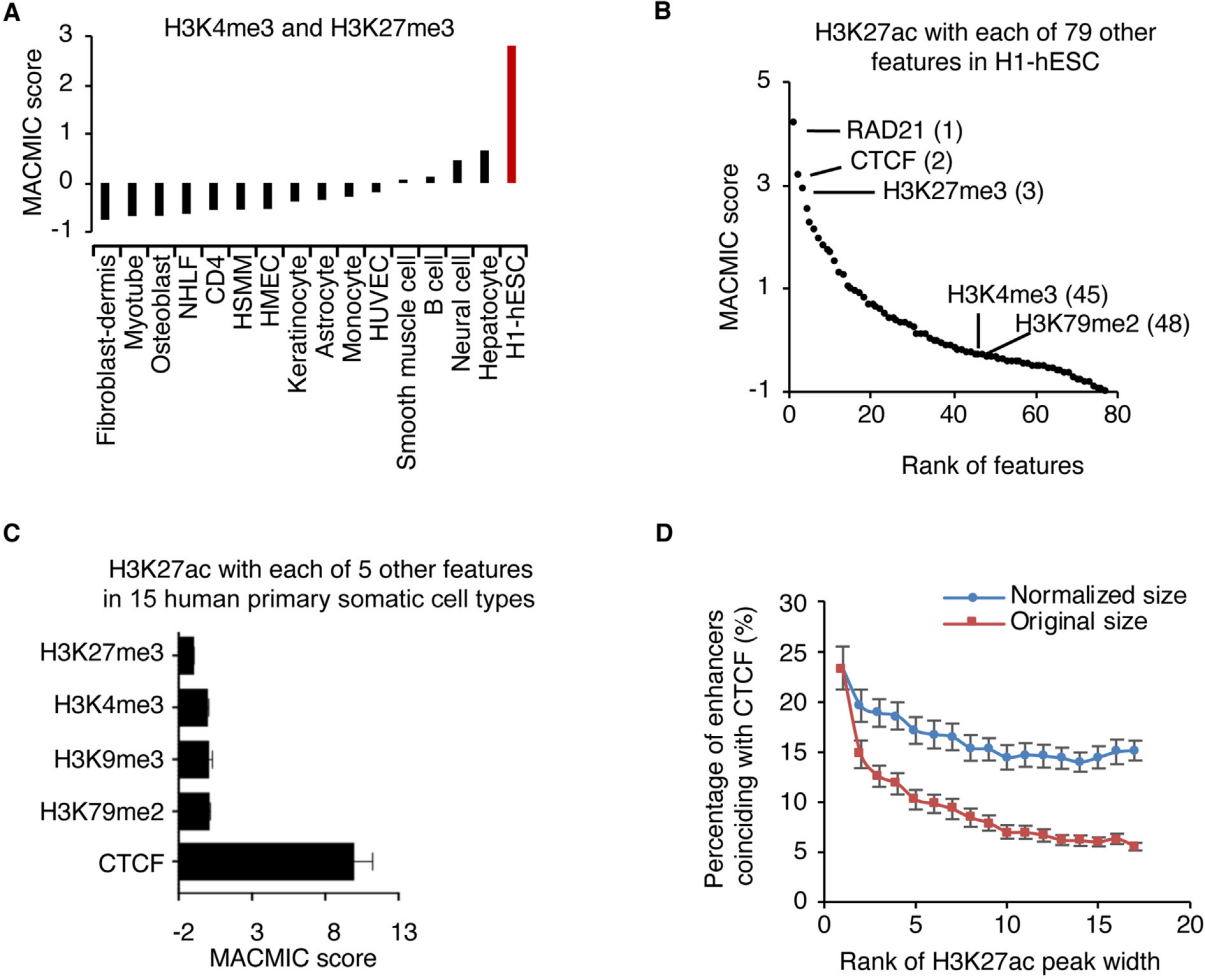
**MACMIC reveals minimal information redundancy of frequent colocalizations between CTCF-binding sites and super-enhancers** **A.** Bar plot to show MACMIC scores between H3K4me3 and H3K27me3 in individual human primary somatic cell types as well as in H1-hESC. **B.** MACMIC scores between H3K27ac and individual other chromatin features in H1-hESC. Number in parentheses indicates the rank of the feature. **C.** MACMIC scores between H3K27ac and individual other chromatin features in 15 human primary somatic cell types. Error bars indicate the standard deviation of MACMIC scores across cell types. **D.** Percentage of enhancers that coincided with CTCF-binding sites in 15 human primary somatic cell types as well as in H1-hESC. Enhancers were divided into individual groups on the base of their H3K27ac width. Each group contains 500 enhancers, *i.e.*, rank 1 contains the widest 500 enhancers, and rank 2 contains the 501st–1000th widest enhancers. NHLF, normal human lung fibroblast; HSMM, human skeletal muscle myoblast; HMEC, human mammary epithelial cell; HUVEC, human umbilical vein endothelial cell.

To further test whether MACMIC scores could effectively recapture feature pairs with biological implications, we analyzed MACMIC scores between H3K4me3 and H3K27me3 in 15 human primary somatic cell types as well as in H1-hESC. In agreement with the reported large number of bivalent domains marked by both H3K4me3 and H3K27me3 in ESCs [30], we observed a large MACMIC score (2.8) in H1-hESC. On the other hand, in agreement with the reported resolution of bivalent domains to form either repressive domains marked by H3K27me3 or active domains marked by H3K4me3 [30], the MACMIC scores between H3K4me3 and H3K27me3 were low in all the 15 primary somatic cell types (from −0.76 to 0.67) (Figure 2A). Therefore, MACMIC analysis successfully recaptured bivalent domains that were known to play a key role in ESCs.

We next tested whether MACMIC analysis could successfully identify new feature pairs that possess a large number of functionally important colocalizations but low correlation. We ranked a set of 79 chromatin features in H1-hESC by the MACMIC scores between the enhancer feature H3K27ac and each of these features (Figure 2B). The top features with the large MACMIC scores in the rank included the suppressive histone modification H3K27me3, consistent with the implication that H3K27ac and H3K27me3 might co-exist in bivalent domains [30]. Interestingly, master regulators of three-dimensional chromatin interaction, the CTCF [31] and its binding partner RAD21 [32], topped in the rank list (Figure 2B). We further performed analysis in 15 human somatic cell types that each had ChIP-seq datasets for a set of 6 chromatin features from the ENCODE project [23] (Table S1). The results showed that the MACMIC score between H3K27ac and the binding of CTCF was significantly larger than MACMIC scores between H3K27ac and the other 4 features including H3K27me3, H3K4me3, H3K9me3, and H3K79me2 (Figure 2C). Moreover, colocalization analysis for CTCF and H3K27ac found that CTCF-binding sites had the largest number of colocalizations with the broadest H3K27ac peaks (super-enhancers) (Figure 2D). To test whether this higher frequency of colocalization was simply due to the longer DNA sequences of super-enhancers, we performed a normalization by lengthening typical enhancers at the two ends of each enhancer, so that the DNA sequences assigned to typical enhancers had equivalent sizes to those of super-enhancers. The result showed that the frequency of colocalization with CTCF-binding sites still tended to be higher for super-enhancers when compared to other enhancers (Figure 2D).

### A unique enrichment of **CSEs** in cell identity genes

**Figure 3:**
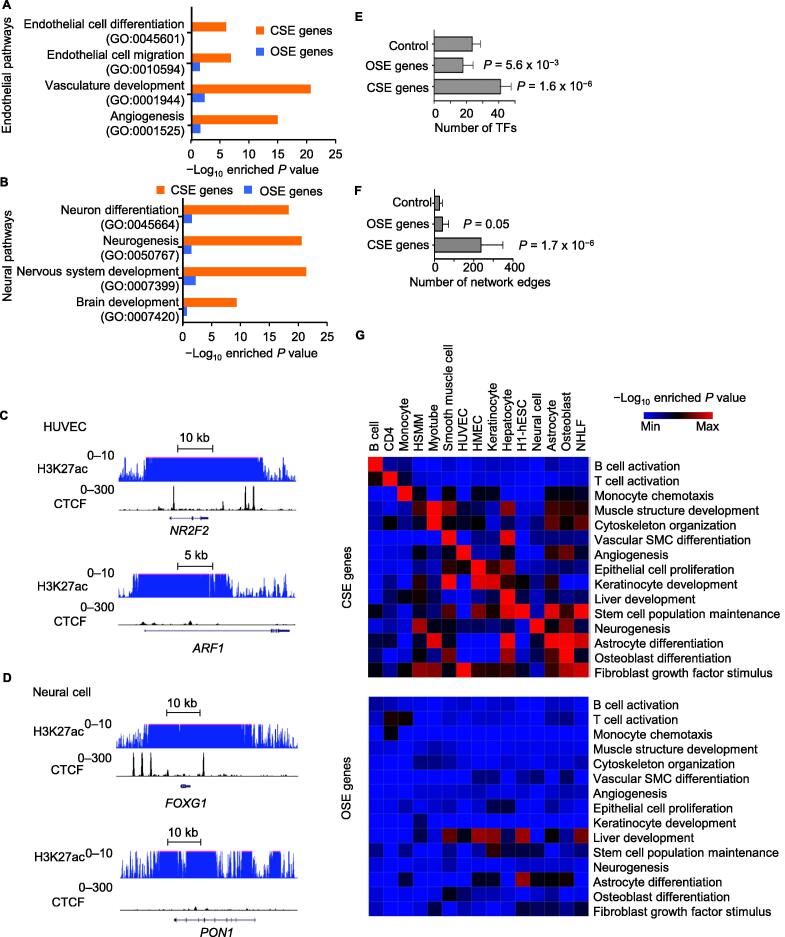
**CSEs mark cell identity genes** **A.** and **B.** Individual pathways enriched in CSE or OSE genes in HUVECs (A) or neural cells (B). **C.** and **D.** ChIP-seq signals for H3K27ac and CTCF at CSE gene *NR2F2* and OSE gene *ARF1* in HUVECs (C) and CSE gene *FOXG1* and OSE gene *PON1* in neural cells (D). **E.** and **F.** The number of transcription factors within each gene group (E) and the number of network edges within each gene group (F) in 15 human primary somatic cell types. Error bars indicate the standard deviation across cell types. Each gene group was defined to have the same number of genes. *P* values were determined by Wilcoxon test in comparison to the control group consisting of randomly selected genes. **G.** Heatmap to show −Log_10_ enriched *P* value of cell type related pathways (rows) in CSE genes (top panel) or OSE genes (bottom panel) defined in each cell type (columns). CSE, CTCF-associated super-enhancer; OSE, other super-enhancer; *NR2F2*, *Nuclear Receptor Subfamily 2 Group F Member 2*; *ARF1*, *ADP Ribosylation Factor 1*; *FOXG1*, *Forkhead Box G1*; *PON1*, *Paraoxonase 1*; TF, transcription factor.

Since super-enhancers were reported to regulate cell identity genes [29], we determined to investigate the role of CTCF in this regulation. We divided super-enhancers into two categories, *i.e.*, CSEs and OSEs. To study the function of genes marked by CSEs and OSEs, we defined the genes of which the gene body overlapped with CSEs or OSEs for at least 1 bp as the  CSE or OSE genes. Intriguingly, only the genes marked by CSEs were significantly enriched in the pathways associated with cell lineage specifications, *e.g.*, the endothelial cell differentiation pathway (GO:0045601) for CSE genes in HUVECs (Figure 3A) and the neuron differentiation pathway (GO:0045664) for CSE genes in neural cells (Figure 3B). Manual inspection of individual known cell lineage factors in these cell types further confirmed the colocalization of ChIP-seq signals of H3K27ac and CTCF, *e.g.*, at the gene *Nuclear Receptor Subfamily 2 Group F Member 2* (*NR2F2*) [33] in HUVECs and the gene *Forkhead Box G1* (*FOXG1*) [34] in neural cells (Figure 3C and D). In contrast, some other genes, although also displaying broad enrichment of H3K27ac, were depleted of CTCF-binding sites, *e.g.*, the gene *ADP Ribosylation Factor 1* (*ARF1*) in HUVECs and the gene *Paraoxonase 1* (*PON1*) in neural cells (Figure 3C and D). Intriguingly, there were typically multiple CTCF-binding sites located within the active region of each CSE. This colocalization pattern was different from the well-known function of CTCF-binding sites as insulators, which often happened between active and repressive domains (Table S4). Besides, a significant portion of the CSE genes encoded transcription factors, whereas we did not observe this phenomenon for the OSE genes (Figure 3E). Further, the CSE genes were connected to a significantly large number of edges in the gene regulatory networks, whereas the numbers of connected network edges were similar for OSE genes and random control genes (Figure 3F). The differences between CSEs and OSEs in their association with genes in cell lineage pathways were highly reproducible in the 15 cell types that we have analyzed (Figure 3G). It was reported that the establishment of cell type specific chromatin loops was important during cell differentiation [35]. Consistently, we found that CSEs were enriched near chromatin loops (Figure S3A) and the boundaries of topologically associating domains (TADs) (Figure S3B), whereas no significant difference in the sizes of the associated TADs was observed between CSEs and OSEs (Figure S3C).

### CSE and OSE genes have similar expression levels and cell type specificities

**Figure 4:**
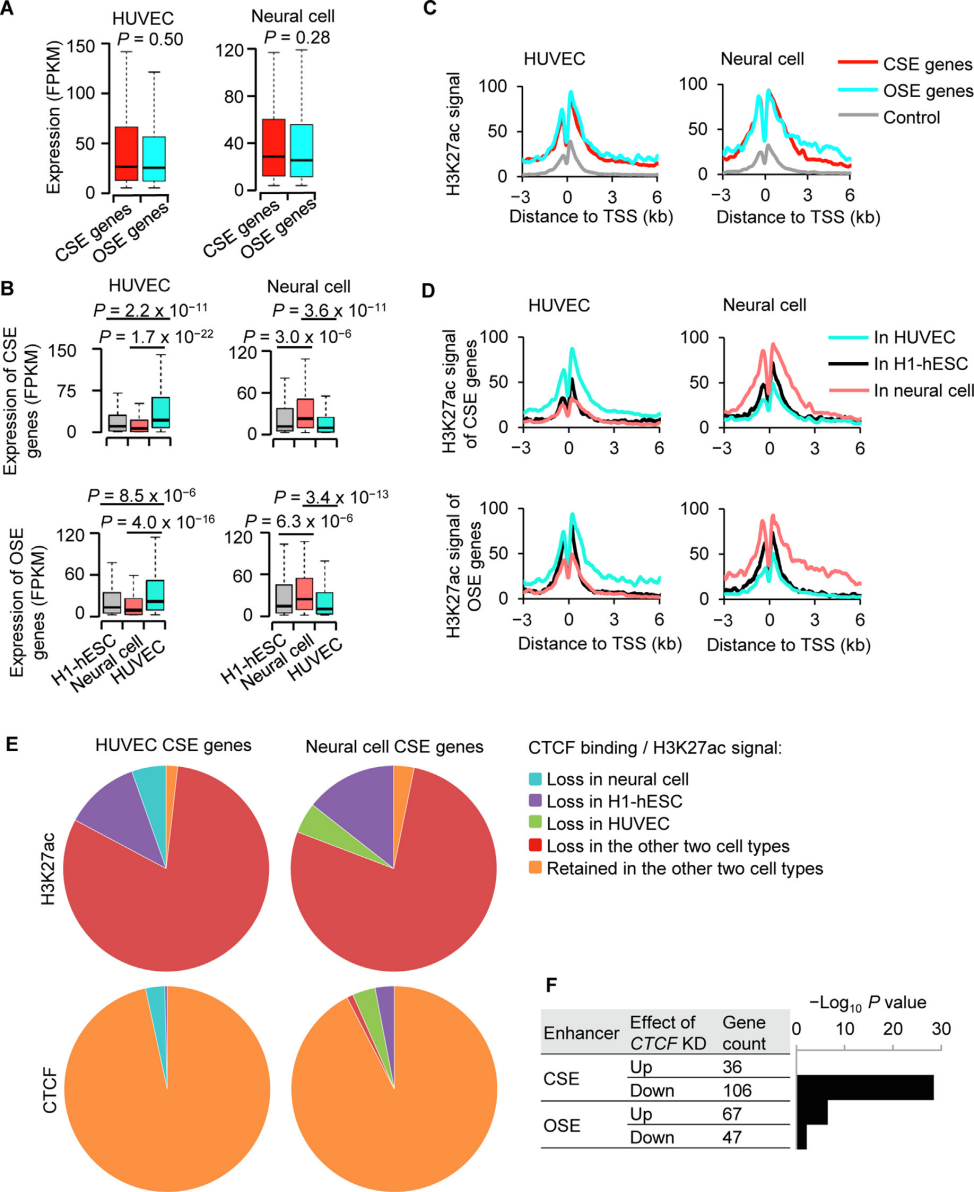
**CTCF is linked to the activation of enhancers** **A.** Box plot to show RNA expression levels of CSE and OSE genes of HUVEC (left panel) and neural cell (right panel) in cell types that defined them. **B**. Box plot to show RNA expression levels of CSE genes (top panels) and OSE genes (bottom panels) in neural cell, HUVEC, and H1-hESC. CSE and OSE genes were defined in HUVEC (left panels) or neural cell (right panels) **C.** H3K27ac signals at CSE gene, OSE genes, and control genes of HUVEC (left panel) and neural cell (right panel) in the cell type that defined these gene groups. **D.** H3K27ac signals at CSE genes (top panels) and OSE genes (bottom panels) in HUVEC, H1-hESC, and neural cells. CSE and OSE genes were defined in HUVEC (left panels) or neural cells (right panels). **E.** Pie charts to show H3K27ac status at HUVEC CSE genes in neural cell and H1-hESC (top left), H3K27ac status at neural cell CSE genes in HUVEC and H1-hESC (top right), binding status of CTCF at HUVEC CSE genes in neural cell and H1-hESC (bottom left), and binding status of CTCF at neural cell CSE genes in HUVEC and H1-hESC (bottom right). **F.** Barplot to show −Log_10_ enriched *P* value of CSE genes or OSE genes in the genes up- or down-regulated by sh*CTCF* in HeLa cells. *P* values were determined by Wilcoxon test in (A), (B), and (F). FPKM, fragments per kilobase million; TSS, transcription start site; KD, knockdown.

To understand how CTCF regulates enhancer activity and in turn regulates cell identity, we first compared the expression levels of genes marked by CSEs and OSEs. Intriguingly, similar expression levels were observed between CSE and OSE genes, and this result was highly reproducible in HUVECs (Figure 4A, left panel) and neural cells (Figure 4A, right panel). Further, CSE and OSE genes of HUVECs were both significantly up-regulated in HUVECs compared to H1-hESCs and neural cells (Figure 4B, left panels). Consistently, CSE and OSE genes of neural cells were both significantly up regulated in neural cells compared to H1-hESCs and HUVECs (Figure 4B, right panels). These results suggested that CSE and OSE genes of the same cell type have similar expression levels and cell type specificities.

We next compared the H3K27ac levels between CSE and OSE genes, as H3K27ac is a marker for enhancer activation. The result indicated that the H3K27ac levels were similar at CSE and OSE genes within HUVECs (Figure 4C, left panel). Similarly, the H3K27ac levels were similar at CSE and OSE genes within neural cells (Figure 4C, right panel). Further, the H3K27ac levels at HUVEC-specific CSE and OSE genes were higher in HUVECs when compared to the same regions in H1-hESCs and neural cells. Similarly, the H3K27ac levels at neuron-specific CSE and OSE genes were higher in neural cells compared to the same regions in HUVECs and H1-hESCs (Figure 4D). Therefore, in agreement with result from the expression analysis, CSE and OSE genes of the same cell type had similar epigenetic states and specificities.

Of the top 500 HUVEC CSE genes, 405 (81%) lost H3K27ac in both neural cells and H1-hESCs (Figure 4E, top left). In contrast, the binding of CTCF in 483 (97%) HUVEC CSE genes were retained in both neural cells and H1-hESCs (Figure 4E, bottom left). Similar results were observed for the neural cell CSE genes. Of the top 500 neural cell CSE genes, 388 (78%) lost H3K27ac in both HUVECs and H1-hESCs (Figure 4E, top right), while the binding of CTCF in 462 (92%) neural cell CSE genes were retained in both HUVECs and H1-hESCs (Figure 4E, bottom right). To further understand the role of CTCF in CSE genes, we next analyzed an RNA-seq dataset from HeLa cells with *CTCF* knocked down or not. The CSE genes of HeLa cells were significantly enriched in the genes down-regulated but not in the genes up-regulated in response to *CTCF* knockdown (Figure 4F). In contrast, the OSE genes showed little enrichment in the down- or up-regulated genes induced by knockdown of *CTCF* (Figure 4F).

Of the top 500 HUVEC OSE genes, 331 (66%) lost H3K27ac in both neural cells and H1-hESCs (Figure S4, top left). In contrast, the binding of CTCF in 492 (98%) HUVEC OSE genes were retained in both neural cells and H1-hESCs (Figure S4, bottom left). Similar results were observed for the neural cell OSE genes. Of the top 500 neural cell OSE genes, 347 (69%) lost H3K27ac in both HUVECs and H1-hESCs (Figure S4, top right), while the binding of CTCF in 476 (96%) neural cell OSE genes were retained in both HUVECs and H1-hESCs (Figure S4, bottom right). These results indicated that although the loss of the activation state of CSEs may not require the loss of CTCF binding, the binding of CTCF was required for the activation of CSEs and their associated genes.

### CSE genes of a given cell type display increased repressive modification H3K27me3 in other cell types

A cell identity gene has two key attributes: 1) it is associated with active chromatin modifications and thus activated to play an important role in the cell type that requires its activation; and 2) it is silenced in most other cell types with repressive chromatin modifications. Since our results demonstrated that the CSE genes of one cell type lost H3K27ac but retained the binding of CTCF in other cell types, we hypothesized that the binding of CTCF might be also important for the repression of these CSE genes in the other cell types.

**Figure 5:**
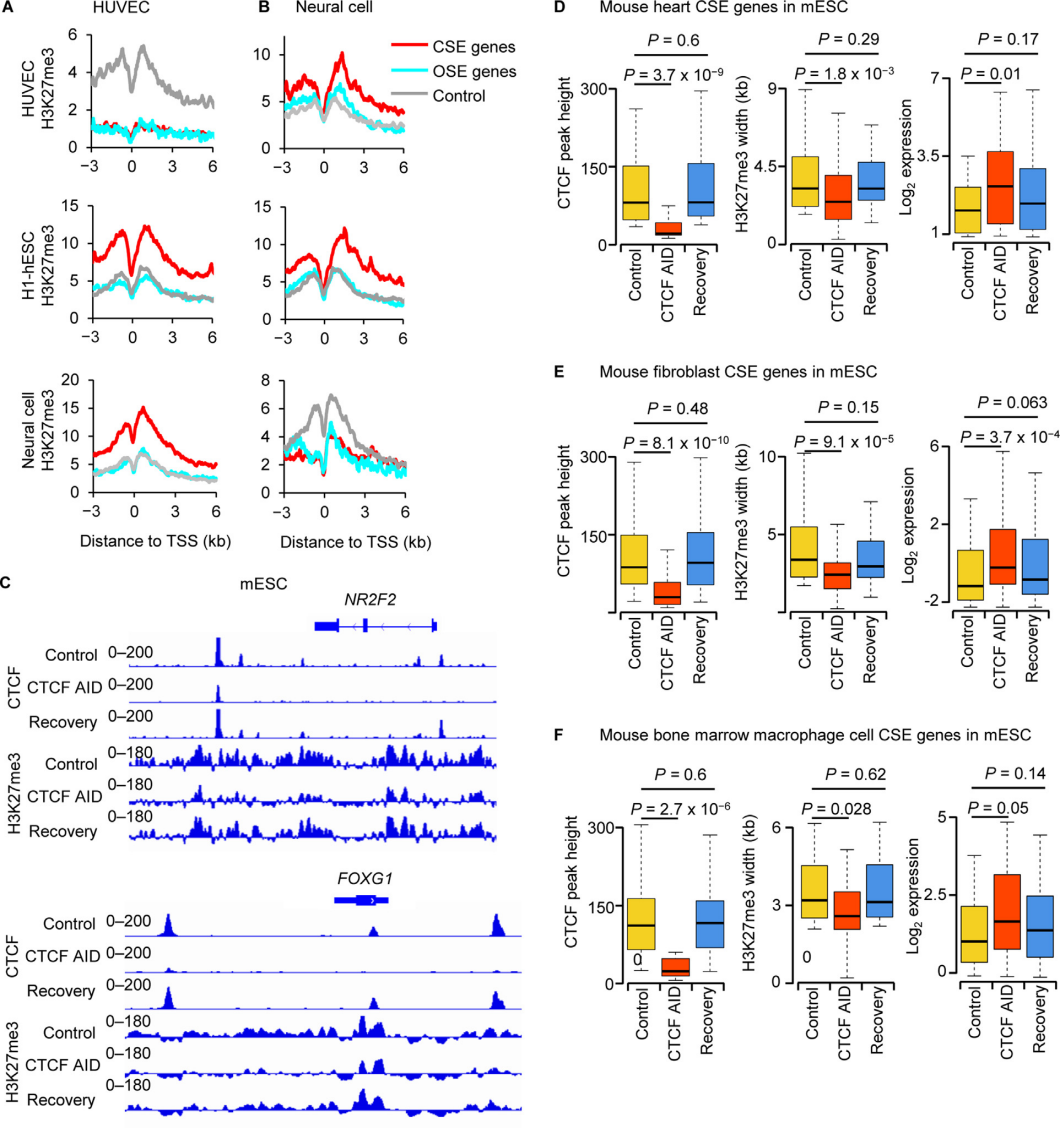
**CTCF regulates cell identity by facilitating the suppressive marker H3K27me3** **A.** and **B.** H3K27me3 signals in H1-hESC, neural cell, and HUVEC at CSE genes, OSE genes, and control genes defined in HUVEC (A) and neural cell (B). **C.** ChIP-seq signals for CTCF and H3K27me3 in mESC at the HUVEC CSE gene *NR2F2* (top) and the neural cell CSE gene *FOXG1* (bottom). **D.**–**F.** Box plot to show the heights of CTCF ChIP-seq enrichment peaks, the widths of H3K27me3 enrichment domains, and the RNA expression levels of CSE genes of mouse heart (D), fibroblast cell (E), and bone marrow macrophage cell (F) under different conditions in mESCs. *P* values were determined by Wilcoxon test. mESC, mouse embryonic stem cell; AID, auxin-inducible degradation.

We first defined a set of CSE genes, a set of OSE genes, and a set of random control genes in HUVECs, and analyzed the pattern of the repressive histone modification H3K27me3 on these three gene sets in each of three cell types including H1-hESCs, neural cells, and also HUVECs. We found that the H3K27me3 signals in HUVECs showed a similar pattern at the HUVEC CSE genes as at the HUVEC OSE genes, and are substantially weaker than the H3K27me3 signals of the random control genes (Figure 5A, top). Intriguingly, only the CSE genes in HUVECs, not those OSE genes in HUVECs or the random control genes, were marked by strong H3K27me3 signals in H1-hESCs (Figure 5A, middle). These trends observed for H3K27me3 in H1-hESCs were the same for H3K27me3 in neural cells (Figure 5A, bottom). Similar results were observed when we defined a set of CSE genes, a set of OSE genes, and a set of random control genes in neural cells to analyze the pattern of H3K27me3 on these three gene sets in HUVECs, H1-hESCs, and neural cells. The H3K27me3 signals in neural cells showed a similar pattern at the neural CSE genes as at the neural OSE genes, but are substantially weaker at the random control genes (Figure 5B, bottom). However, only the CSE genes of neural cells, not the OSE genes of neural cells or the random control genes, possessed strong H3K27me3 signals in H1-hESCs (Figure 5B, middle). These trends observed for H3K27me3 in H1-hESCs were the same for H3K27me3 in HUVECs (Figure 5B, top).

We next further included another 13 sets of biosamples that each had ChIP-seq data for CTCF, H3K27ac, and H3K27me3. Consistent with the results from HUVECs and neural cells, CSE and OSE genes showed similar enrichment of H3K27ac (Figure S5A) and similar depletion of H3K27me3 (Figure S5B) in cell types that defined these CSE and OSE genes. Next, we analyzed these CSE and OSE genes in H3K27ac ChIP-Seq datasets from 84 biosamples and H3K27me3 ChIP-seq datasets from 125 biosamples from the ENCODE database. CSE and OSE genes both showed attenuated enrichment of H3K27ac when the H3K27ac was analyzed in cell types different from the cell types that defined the CSE and OSE genes (Figure S5C). However, the CSE genes were associated with significant enrichment of H3K27me3, whereas the OSE genes showed little enrichment of H3K27me3, when the H3K27me3 was analyzed in cell types different from the cell types that defined these CSE and OSE genes (Figure S5D). These analyses indicated that the CSE genes, but not the OSE genes, were under stringent epigenetic repression by H3K27me3 in cell types different from the cell types that defined the CSE and OSE genes. Interestingly, CTCF and H3K27me3 are also among the top feature pairs ranked by MACMIC score in H1-hESC (Figure S6).

### **CTCF** in a given cell type is required for the repression of CSE genes defined in other cell types

Due to limited availability of public datasets for human, we analyzed the mouse homologs of CSE and OSE genes defined in HUVECs and human neural cells in mESCs with CTCF ChIP-seq and H3K27me3 ChIP-seq data under normal and auxin-inducible degradation (AID) conditions. Importantly, auxin-induced degradation of CTCF in mESCs led to the loss of CTCF binding and H3K27me3 signals in mESCs at the mouse homologs of CSE genes defined in HUVECs and human neural cells. For example, signals of CTCF binding and H3K27me3 in mESCs at known identity genes of somatic cell types, the *NR2F2* [33] of endothelial cells (Figure 5C, top) and the *FOXG1* [34] of neural cells (Figure 5C, bottom), were substantially attenuated after auxin-induced degradation of CTCF, and recovered after auxin was washed off (Figure 5C). The CTCF-binding sites in mESCs at these CSE genes were located within the broad H3K27me3 modifications. To further validate our results, we used ChIP-seq data for CTCF and H3K27ac in three mouse primary samples including heart, fibroblast cell, and bone marrow macrophage to define CSE and OSE genes, and analyzed CTCF and H3K27me3 at these genes in mESCs. The results showed that the colocalization of CTCF-binding sites and broad H3K27me3 in mESCs was similar to the colocalization observed for CTCF-binding sites and super-enhancers in mouse heart, fibroblast cell, and bone marrow macrophage. Our further analysis indicated that in parallel with the loss of CTCF binding in mESCs at the CSE genes of mouse heart, fibroblast cell, and bone marrow macrophage, the H3K27me3 signals in mESCs were reduced dramatically and the expression levels in mESCs were significantly up-regulated (Figure 5D–F). Taken together, these results suggested that the CTCF in a given cell type was required for the repression of CSE genes defined in a different cell type.

## Discussion

Conventional analysis of relationship between chromatin features tends to focus on strongly positive or negative correlation to identify the associated components within a specific biological process [1]. However, genomic features with weak correlation across the genome may still colocalize at many genomic sites in a biologically important manner. It is hard to capture the significance of such colocalizations on the basis of conventional correlation analysis. In this study, we provide a new method to identify MACMIC, which effectively prioritizes the feature pairs with low genome-wide correlation but substantial colocalizations. Using the MACMIC, we successfully recapture the reported bivalent domains in ESCs, which is composed of both activating histone modifications, *e.g.*, H3K4me3, and the repressive histone modifications, *e.g.*, H3K27me3. Activating histone modification and the repressive histone modification possess low genome-wide correlation in the ESCs, but the colocalizations of them at bivalent domains mark important lineage specific regulators.

As proof of principle, we present a novel relationship identified by MACMIC between the binding of CTCF and the enhancer marker H3K27ac. Our analysis demonstrated that their colocalization is key to both the activation and repression of cell identity genes. Numerous efforts have been made to understand cell identity regulation [26]. Somatic cells, such as fibroblasts [36], keratinocytes [37], peripheral blood cells [38], and neural progenitor cells [39], have been sucessfully reprogrammed to induced pluripotent stem cells. Many transcription factors and epigenetic regulators have been proposed to play important roles in these dynamic processes. We and several other groups recently discovered that cell identity genes manifested unique chromatin epigenetic signatures associated with their distinct transcriptional regulation mechanisms [29], [40], [41], [42]. CTCF is well known for its function as an insulator that binds regions between active and repressive domains on chromatin [43], as a mediator for promoter–enhancer interaction [44], and as a partner of cohesin in regulating chromatin 3D structure [45], [46]. It further has been proven to be an essential factor for cell differentiation and development of T cell [47], neuron [48], heart [49], and limb [50]. However, how these functions of CTCF are connected to the regulation of cell identity genes is not known.

In this study, we separate CSEs from OSEs based on the colocalization of CTCF-binding sites with H3K27ac signals in CSEs. Our results suggest that CTCF contributes to the activation of CSE genes in cell types that require the activation to define their specific lineage. These same CSE genes are repressed in other cell types, whose repression also requires CTCF colocalizing with H3K27me3 signals. Interestingly, only CSE genes (but not OSE genes) showed significantly higher H3K27me3 signals in the cell types that required their repression. This observation is consistent with the notion that cell identity genes of a different lineage must be epigenetically repressed in other somatic cell types (Figure 5). In response to the loss of CTCF function in ESCs, H3K27me3 signals in ESCs at the CSE genes of somatic cell types were dramatically reduced but restored after recovery of CTCF function (Figure 5). Intriguingly, the CTCF-binding sites in ESCs at somatic cell identity genes were located within their repressive domains in ESCs. This colocalization was similar to the colocalization of CTCF-binding sites with super-enhancers observed in somatic cell types. These unique CTCF-associated epigenetic profiles suggested a novel function of CTCF in epigenetic regulation of transcription.

Recently, many epigenetic regulators have been proven to interact with CTCF in different biological processes. For instance, Bromodomain Containing 2 (BRD2) has been reported to directly interact with CTCF during Th17 cell differentiation [51]. This report suggested that CTCF might be able to regulate enhancer signals by facilitating the binding of enhancer mediators on the chromatin [52]. Interestingly, our result indicates that CTCF plays an important role for the repressive histone modification, H3K27me3. A recent study has reported that depletion of CTCF does not affect the spreading of H3K27me3 [53], indicating that CTCF might affect H3K27me3 modification by a process other than the spreading. Considering that CTCF has been reported to regulate *Insulin-like growth factor II* (*Igf2*) expression by direct interaction with SUZ12 Polycomb Repressive Complex 2 Subunit (Suz12), an important component of Polycomb repressive complexe 2 (PRC2) [54], it is possible that CTCF may serve as a landmark to facilitate the localization of epigenetic regulators.

Interestingly, among the top-ranked feature pairs in H1-hESC, there are many pairs that are formed by a factor associated with chromatin structure and a factor associated with histone modification for transcription activation or repression. For example, we observed the combination of RB Binding Protein 5 (RBBP5) [55] and RAD21 [32] and the combination of Lysine-specific Demethylase 4A (KDM4A) [32] and RAD21. RBBP5 and KDM4A are important regulators of H3K4me3, and RAD21 is a component of the cohesion complex that regulates chromatin looping. In addition, we further observed additional combinations that each includes a factor associated with transcription activation and a factor associated with transcription repression, such as C-terminal Binding Protein 2 (CTBP2) [56] and H3K27ac. This kind of combination is consistent with the concept of bivalent domains in stem cells. Last but not the least, we found high-score combinations that each includes a factor of the cohesion complex and a factor associated with transcription repression, such as the combination of CTCF and H3K27me3, which we found later is also very important for the cell identity regulation.

Taken together, through MACMIC analysis, we find that CTCF plays an important role in the epigenetic regulation of cell identity. Further analysis suggests that CTCF is important for the regulation of both enhancer signals and repressive signals at the CSE genes in a cell-type specific manner. Although our analysis focused on the colocalization of enhancer signal with the other chromatin feature, MACMIC analysis has great potential to identify many other novel biologically significant colocalizations between chromatin features that have low global correlation across the genome. With the increased usage of sequencing technologies, more potential feature pairs can be identified. This will provide opportunities in the future to further understand the function of chromatin in transcription, replication, DNA repair, and many other biological processes.

## Code availability

The code for MACMIC is available at the website GitHub, https://github.com/bxia888/MACMIC.

## CRediT author statement

**Guangyu Wang:** Investigation, Methodology, Software, Resources, Visuali, zation, Validation, Data curation, Formal analysis, Writing - original draft, Writing - review & editing. **Bo Xia:** Investigation, Methodology, Software, Resources, Visualization, Validation, Data curation, Formal analysis, Writing - original draft, Writing - review & editing. **Man Zhou:** . **:** Investigation, Resources, Writing - review & editing. **Jie Lv:** Investigation, Resources, Writing - review & editing. **Dongyu Zhao:** Investigation, Resources, Writing - review & editing. **Yanqiang Li:** Investigation, Resources, Writing - review & editing. **Yiwen Bu:** Investigation, Resources, Writing - review & editing. **Xin Wang:** Investigation, Resources, Writing - review & editing. **John P. Cooke:** Funding acquisition, Resources, Writing - review & editing. **Qi Cao:** Funding acquisition, Resources, Writing - review & editing. **Min Gyu Lee:** Funding acquisition, Resources, Writing - review & editing. **Lili Zhang:** Investigation, Resources, Writing - review & editing. **Kaifu Chen:** Conceptualization, Supervision, Project administration, Funding acquisition, Resources, Investigation, Methodology, Visualization, Validation, Data curation, Formal analysis, Writing - original draft, Writing - review & editing. All authors read and approved the final manuscript.

## Competing interests

The authors have no competing interest that might influence the performance or presentation of the work described in this manuscript.

## Supplementary material

Supplementary Figure S1 **Colocalizations of globally uncorrelated features reveal unique functional pathways A.** and **B.** Scatter plot of H3K4me3 width and H3K27ac width (A) and H3K4me3 width and H3K27me3 width (B) in H1-hESC. **C.** Venn diagram to show overlap between genes associated with broad H3K4me3, broad H3K27ac, and colocalization of broad H3K4me3 and broad H3K27ac. **D.** Venn diagram to show overlap between genes associated with broad H3K4me3, broad H3K27me3, and colocalization of broad H3K4me3 and broad H3K27me3. **E.** Heatmap to show width of H3K4me3, H3K27ac, and H3K27me3 at individual genes from different groups. **F.** Heatmap to show −log_10_ enrichment *P* values of GO terms in genes associated with broad H3K4me3, broad H3K27me3, and colocalization of broad H3K4me3 and broad H3K27me3. **G.** Bar plot to show −log_10_ enrichment *P* values of example pathways identified by colocalization of broad H3K4me3 and broad H3K27me3 in H1-hESC.

Supplementary Figure S2 **The MACMIC method to define information redundancy of colocalizations between genomic features with different methods A.** Scatter plot to show 1-PCA values and the number of colocalizations of each of 225 feature pairs derived from 6 features in 15 human primary cell types. **B.** Scatter plot to show R2 values and the number of colocalizations of each of 225 feature pairs derived from 6 features in 15 human primary cell types. **C.** Scatter plot to show MACMIC scores and 1- PCA values of each of 225 feature pairs derived from 6 features in 15 human primary cell types. **D.** Scatter plot to show MACMIC scores and R^2^ of each of 225 feature pairs derived from 6 features in 15 human primary cell types.

Supplementary Figure S3 **CSEs are located close to the anchor sites of chromatin loops and the boundaries of topologically associating domains in HUVECs A.** Box plot to show the distances between the anchor sites of chromatin loops and CTCF associated super-enhancers (CSEs) or other super-enhancers (OSEs). **B.** Box plot to show the distances between the boundaries of topologically associating domains and CSEs or OSEs. **C.** Box plot to show the sizes of TADs associated with CSEs or OSEs. *P* values were determined by Wilcoxon test.

Supplementary Figure S4 **Statuses of H3K27ac and****CTCF****binding at HUVEC/neural cell OSE-marked genes****in HUVEC****,****n****eural****c****ell****, and H1-hESC** Pie charts to show H3K27ac status at HUVEC OSE-marked genes in neural cells and H1-hESCs (top left), H3K27ac status at neural cell OSE-marked genes in HUVECs and H1-hESC (top right), binding status of CTCF at HUVEC OSE-marked genes in neural cells and H1-hESCs (bottom left), and binding status of CTCF at neural cell OSE-marked genes in HUVECs and H1-hESCs (bottom right).

Supplementary Figure S5 **CSE-marked genes****in a cell type is linked to the****increased repressive modification H3K27me3****in other cell types A.** and **B.** Box plot to show H3K27ac width (A) and H3K27me3 width (B) at CSE- or OSE-marked genes in cell types that defined these CSE- and OSE-marked genes. Each boxplot combined data from 15 human primary somatic cell types. **C.** and **D.** Box plot to show H3K27ac width (C) in 84 biosamples and H3K27me3 width (D) in 125 biosamples from ENCODE database at CSE- or OSE-marked genes defined in each of 15 human primary somatic cell types.

Supplementary Figure S6 **MACMIC scores of different feature pairs in H1-hESC** X-axis indicates rank of MACMIC scores for individual feature pairs.
