# Supplementary figures and images for: MACMIC Reveals A Dual Role of CTCF in Epigenetic Regulation of Cell Identity Genes
Source: Genomics Proteomics Bioinformatics. 2021 Mar 5;19(1):140–53. doi: 10.1016/j.gpb.2020.10.008 (PMC8498966; doi:10.1016/j.gpb.2020.10.008)

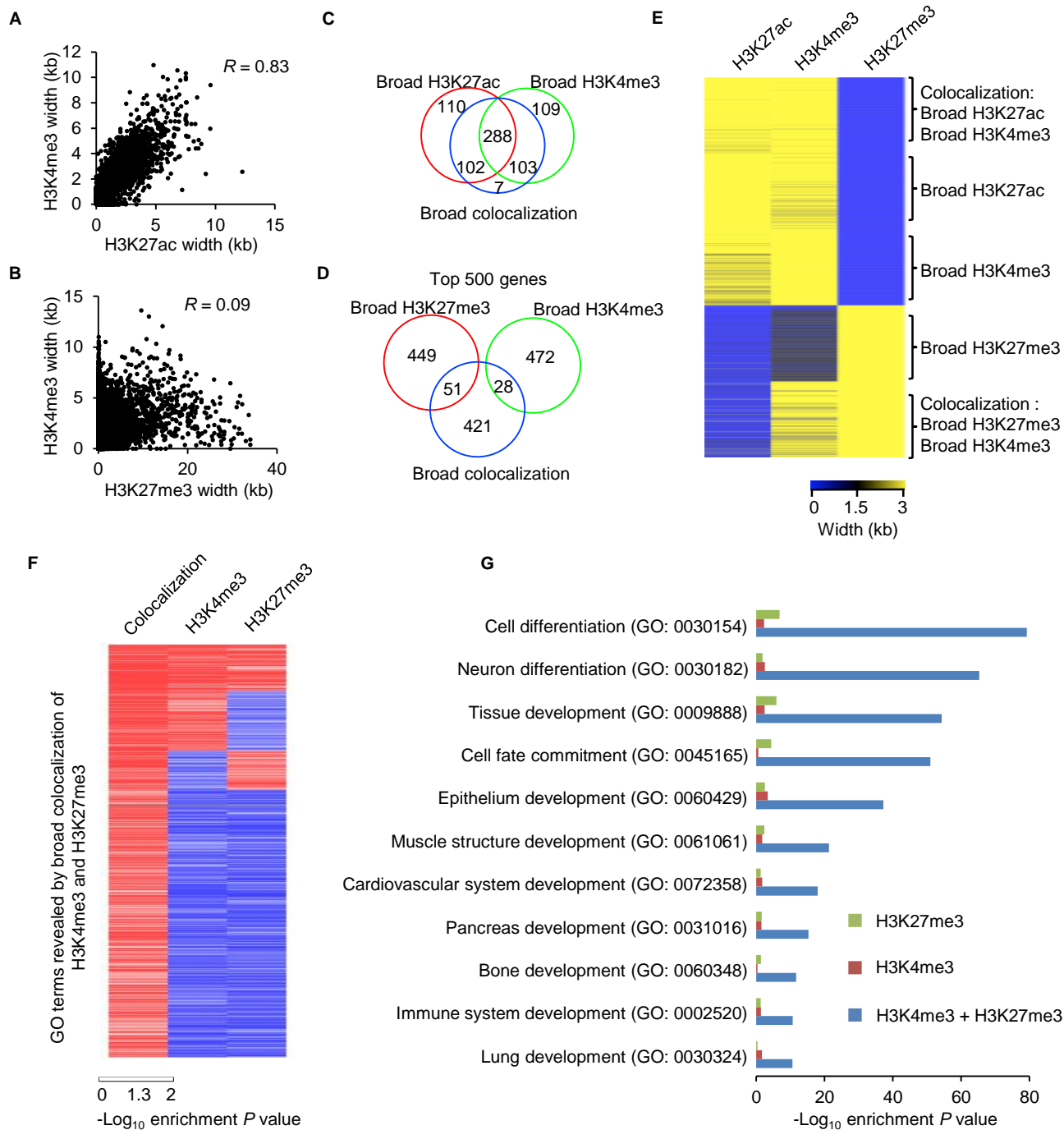

Supplement: Supplementary Figure S1 — Colocalizations of globally uncorrelated features reveal unique functional pathways A. and B. Scatter plot of H3K4me3 width and H3K27ac width (A) and H3K4me3 width and H3K27me3 width (B) in H1-hESC. C. Venn diagram to show overlap between genes associated with broad H3K4me3, broad H3K27ac, and colocalization of broad H3K4me3 and broad H3K27ac. D. Venn diagram to show overlap between genes associated with broad H3K4me3, broad H3K27me3, and colocalization of broad H3K4me3 and broad H3K27me3. E. Heatmap to show width of H3K4me3, H3K27ac, and H3K27me3 at individual genes from different groups. F. Heatmap to show −log10 enrichment P values of GO terms in genes associated with broad H3K4me3, broad H3K27me3, and colocalization of broad H3K4me3 and broad H3K27me3. G. Bar plot to show −log10 enrichment P values of example pathways identified by colocalization of broad H3K4me3 and broad H3K27me3 in H1-hESC. [file mmc1.pdf]

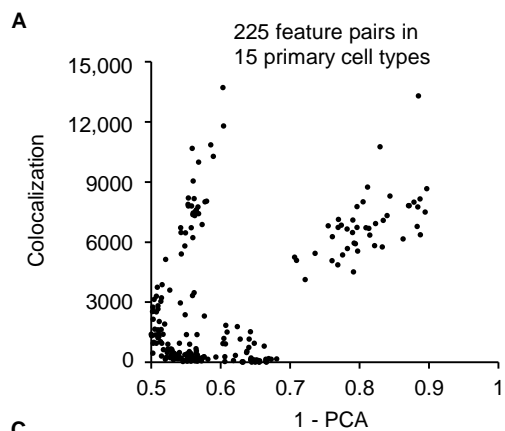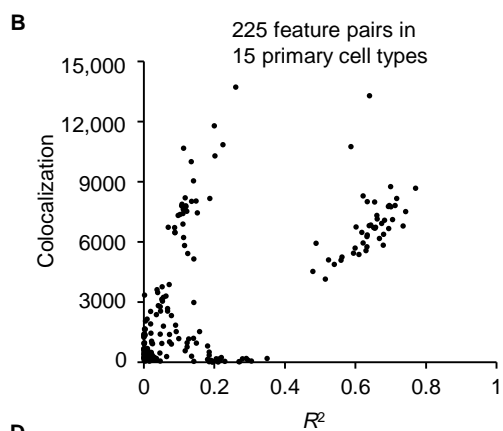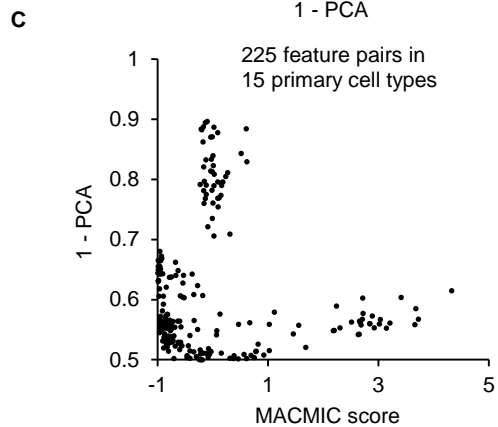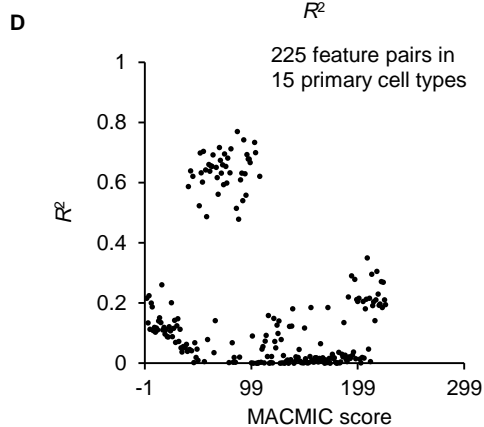

Supplement: Supplementary Figure S2 — The MACMIC method to define information redundancy of colocalizations between genomic features with different methods A. Scatter plot to show 1-PCA values and the number of colocalizations of each of 225 feature pairs derived from 6 features in 15 human primary cell types. B. Scatter plot to show R2 values and the number of colocalizations of each of 225 feature pairs derived from 6 features in 15 human primary cell types. C. Scatter plot to show MACMIC scores and 1- PCA values of each of 225 feature pairs derived from 6 features in 15 human primary cell types. D. Scatter plot to show MACMIC scores and R2 of each of 225 feature pairs derived from 6 features in 15 human primary cell types. [file mmc2.pdf]

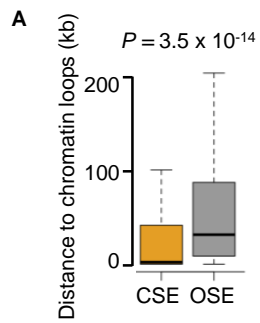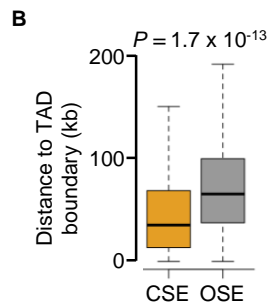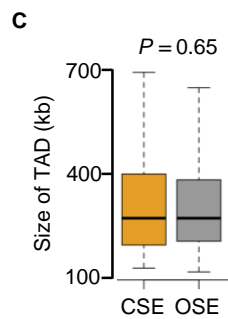

Supplement: Supplementary Figure S3 — CSEs are located close to the anchor sites of chromatin loops and the boundaries of topologically associating domains in HUVECs A. Box plot to show the distances between the anchor sites of chromatin loops and CTCF associated super-enhancers (CSEs) or other super-enhancers (OSEs). B. Box plot to show the distances between the boundaries of topologically associating domains and CSEs or OSEs. C. Box plot to show the sizes of TADs associated with CSEs or OSEs. P values were determined by Wilcoxon test. [file mmc3.pdf]

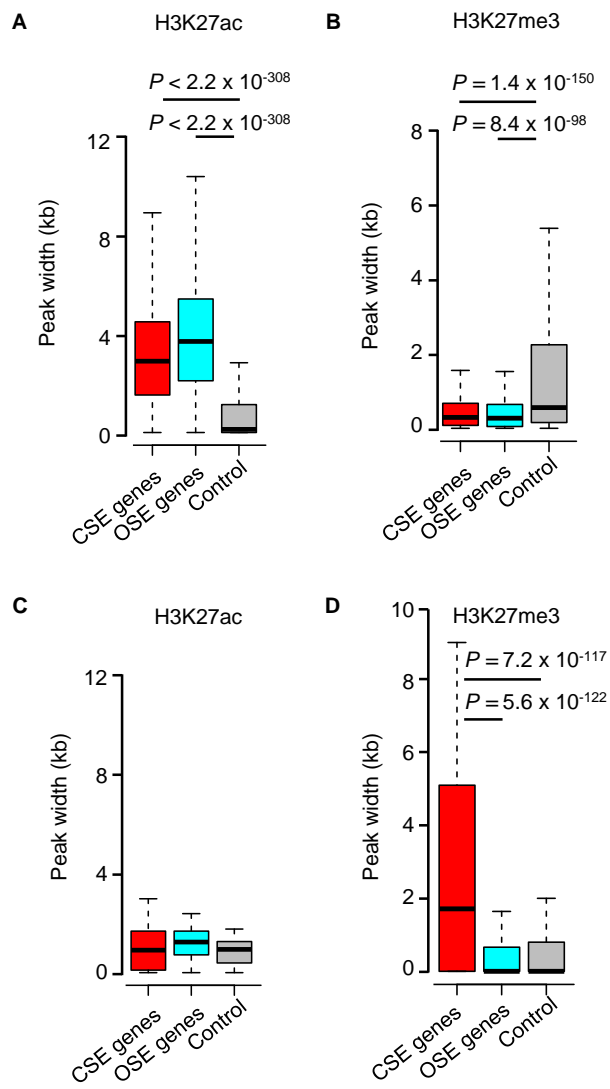

Supplement: Supplementary Figure S5 — CSE-marked genesin a cell type is linked to theincreased repressive modification H3K27me3in other cell types A. and B. Box plot to show H3K27ac width (A) and H3K27me3 width (B) at CSE- or OSE-marked genes in cell types that defined these CSE- and OSE-marked genes. Each boxplot combined data from 15 human primary somatic cell types. C. and D. Box plot to show H3K27ac width (C) in 84 biosamples and H3K27me3 width (D) in 125 biosamples from ENCODE database at CSE- or OSE-marked genes defined in each of 15 human primary somatic cell types. [file mmc5.pdf]

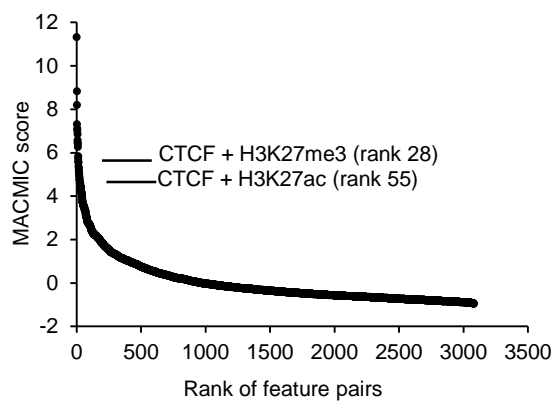

Supplement: Supplementary Figure S6 — MACMIC scores of different feature pairs in H1-hESC X-axis indicates rank of MACMIC scores for individual feature pairs. [file mmc6.pdf]
