# Supplementary material for: MACMIC Reveals A Dual Role of CTCF in Epigenetic Regulation of Cell Identity Genes
Source: Genomics Proteomics Bioinformatics. 2021 Mar 5;19(1):140–53. doi: 10.1016/j.gpb.2020.10.008 (PMC8498966; doi:10.1016/j.gpb.2020.10.008)
Supplement: Supplementary Figure S4 — Statuses of H3K27ac andCTCFbinding at HUVEC/neural cell OSE-marked genesin HUVEC,neuralcell, and H1-hESC Pie charts to show H3K27ac status at HUVEC OSE-marked genes in neural cells and H1-hESCs (top left), H3K27ac status at neural cell OSE-marked genes in HUVECs and H1-hESC (top right), binding status of CTCF at HUVEC OSE-marked genes in neural cells and H1-hESCs (bottom left), and binding status of CTCF at neural cell OSE-marked genes in HUVECs and H1-hESCs (bottom right). [file mmc4.pdf]

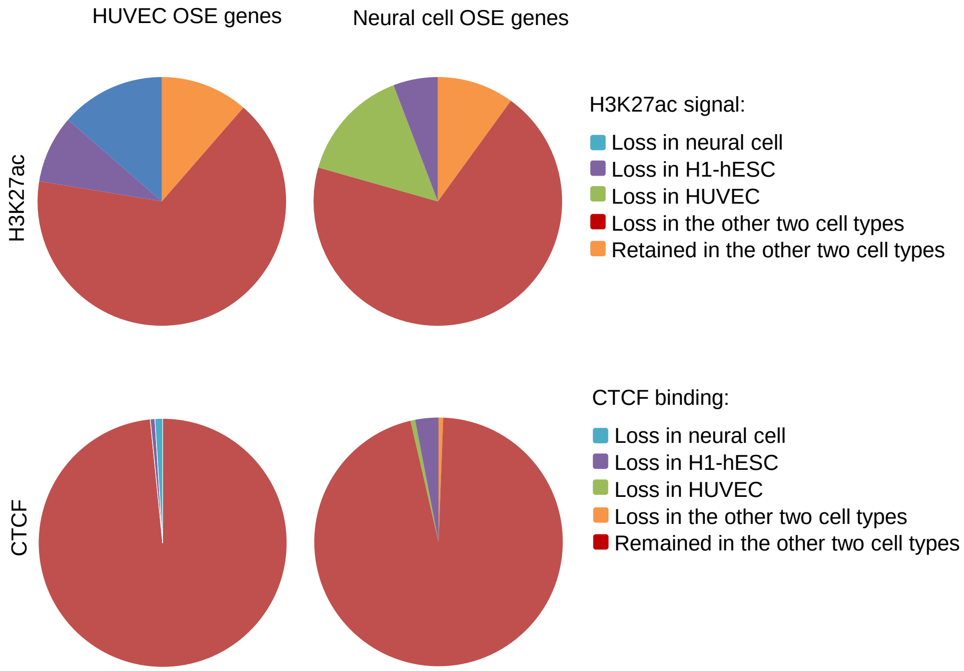

HUVEC OSE genes
Neural cell OSE genes
H3K27ac
H3K27ac signal:
Loss in neural cell
Loss in H1-hESC
Loss in HUVEC
Loss in the other two cell types
Retained in the other two cell types
CTCF
CTCF binding:
Loss in neural cell
Loss in H1-hESC
Loss in HUVEC
Loss in the other two cell types
Remained in the other two cell types
