# Supplementary material for: MACMIC Reveals A Dual Role of CTCF in Epigenetic Regulation of Cell Identity Genes
Source: Genomics Proteomics Bioinformatics. 2021 Mar 5;19(1):140–53. doi: 10.1016/j.gpb.2020.10.008 (PMC8498966; doi:10.1016/j.gpb.2020.10.008)
Supplement: Supplementary Table S2 [file mmc8.docx]

**Table S2 GEO datasets analyzed in this paper**

| Dataset | Description |
| --- | --- |
| GSE98671 | Auxin induced degradation of *CTCF* |
| GSE38495 | H1-hESC single cell |
| GSE51254 | HCT116 single cell |
| GSE108869 | shCTCF in HeLa cell |
| GSE99518 | mESC *YY1* ChipSeq |
| GSE30203 | mESC *CTCF* ChipSeq |
| GSE62380 | mESC H3K27ac ChipSeq |
| GSE44286 | mESC H3K4me3 ChipSeq |
| GSE11172 | mESC H3K4me1 ChipSeq |
| GSE30919 | mESC H3K27me3 ChipSeq |
| GSE24165 | mESC H3K27ac ChipSeq |
| GSE112806 | mESC enhancer mediators |
| GSE63525 | HUVEC HiC |

Note: GEO accession numbers that are used to perform analysis in this manuscript.
